# Comparative efficacies of different antibiotic treatments to eradicate nontypeable *Haemophilus influenzae *infection

**DOI:** 10.1186/1471-2334-8-15

**Published:** 2008-02-07

**Authors:** Yukie Sekiya, Masahiro Eguchi, Masahiko Nakamura, Kimiko Ubukata, Satoshi Omura, Hidenori Matsui

**Affiliations:** 1 Center for Basic Research, The Kitasato Institute, 5-9-1 Shirokane, Minato-ku, Tokyo 108-8642, Japan; 2 Kitasato Institute for Life Sciences and Graduate School of Infection Control Sciences, 5-9-1 Shirokane, Minato-ku, Tokyo 108-8641, Japan; 3 Center for Clinical Pharmacy and Clinical Sciences, School of Pharmaceutical Sciences, 5-9-1 Shirokane, Minato-ku, Tokyo 108-8641, Japan

## Abstract

**Background:**

Nonencapsulated and nontypeable *Haemophilus influenzae *(NTHi) is a major cause of human respiratory tract infections. Some strains of NTHi can cause invasive diseases such as septicemia and meningitis, even if *H. influenzae *is not generally considered to be an intracellular pathogen. There have been very few reports about the therapeutic efficacy of antibiotics against respiratory tract infection caused by NTHi in mice because it is difficult for *H. influenzae *to infect mice. Therefore, we evaluated the efficacy of antibiotics against NTHi in both a cell culture model and a mouse model of infection.

**Methods:**

We used six strains of NTHi isolated from adult patients with chronic otitis media, namely three β-lactamase-negative ampicillin (AMP)-resistant (BLNAR) strains and three β-lactamase-negative AMP-susceptible (BLNAS) strains, to evaluate the efficacy of AMP, cefcapene (CFPN), levofloxacin (LVX), clarithromycin (CLR), and azithromycin (AZM) in both a cell culture infection model and a mouse infection model. In the cell culture infection model, strains that invade A549 human alveolar epithelial cells were treated with each antibiotic (1 μg/ml). In the mouse infection model, female C57BL/6 mice were intraperitoneally injected with cyclophosphamide (200 mg/kg) three days before intranasal infection with 1 × 10^9 ^colony-forming units (CFU) of NTHi and on the day of infection. After infection, the mice were orally administered each antibiotic three times daily for three days, except for AZM, which was administered once daily for three days, at a dose of 100 mg/kg/day.

**Results:**

In the cell culture infection model, it was found that two BLNAR strains were able to enter the cell monolayers by the process of macropinocytosis, and treatment with LVX yielded good bactericidal activity against both strains inside the cells. In the mouse infection model, no bacteria were detected by means of plating the lung homogenates of LVX-treated mice at day 4 after infection, while more than 10^5 ^CFU of bacteria per tissue sample were detected in nontreated mice.

**Conclusion:**

Our findings show the outcome and rich benefits of fluoroquinolone treatment of respiratory infections caused by either invasive or noninvasive BLNAR strains of NTHi.

## Background

*Haemophilus influenzae*, a species that can cause systemic disease in humans, is an obligate human commensal found principally in the upper respiratory tract [1]. *H. influenzae *can express one or none of six antigenically distinct polysaccharide capsules [1]. *H. influenzae *type b (Hib) penetrates the nasopharyngeal mucosa of its host by a presently unknown mechanism [2]. An *in vitro *invasion assay showed that Hib has a very weak propensity to enter cultured epithelial cells [2]. In contrast, a decapsulated mutant of Hib was found to be consistently 20 to 100 times more invasive than its wild type [2]. Moreover, nonencapsulated and nontypeable *H. influenzae *(NTHi) are consistently over 1,000 times more invasive than Hib [2]. Some strains of NTHi can cause invasive diseases such as septicemia and meningitis, which suggests that the bacteria can pass through the cell layers and survive within both epithelial and endothelial cells [3]. Actually, it has been reported that NTHi can pass through viable cell layers of the human lung epithelial cell line NCI-H292 by paracytosis, thus requiring bacterial protein synthesis, even though the passage time is dependent on the growth rate, which is influenced by the bacterial strains [4]. Although NTHi is a major cause of human infections at present, to the best of our knowledge only one comparative evaluation of antibiotics using an NTHi infection model in mice has been reported [5]. As a matter of fact, mouse models of infection are not typical for *H. influenzae *because it is difficult for this bacterium to infect mice via the respiratory route.

In the present study, we utilized the A549 human lung epithelial cell line to characterize the invasive phenomena of six clinically isolated strains of NTHi, namely three β-lactamase-negative ampicillin (AMP)-resistant (BLNAR) strains and three β-lactamase-negative AMP-susceptible (BLNAS) strains. Two invasive strains were identified among these six strains, and the invasive strains were then used to evaluate the efficacy of antibiotics including AMP, cefcapene (CFPN), levofloxacin (LVX), clarithromycin (CLR), and azithromycin (AZM) in a cell culture model of infection. In addition, the efficacy of each antibiotic was also evaluated against each of the six isolated strains in an established mouse model of chronic pulmonary infection with the required chemically induced damage before infection.

We show that LVX was the most effective antibiotic against each NTHi strain across both cell culture and mouse infection models.

## Methods

### Study design

This study was approved by the Ethics Committee on Investigations of Experimental Pain in Conscious Animals, Kitasato Institute for Life Sciences, Kitasato University, which followed internationally recognized guidelines.

### Bacterial strains and culture conditions

We used six NTHi strains isolated in 2005 from adult patients with otitis media in Japan (age range, 24–68 years; mean age, 40.5 years): three strains of BLNAR (YS001, YS002, and YS005) for which the AMP minimum inhibitory concentration (MIC) was ≥ 1.0 μg/ml, and three strains of BLNAS (YS008, YS009, and YS010). Bacteria were grown without shaking in brain heart infusion (BHI) broth (Becton Dickinson and Company, Sparks, MD, USA) supplemented with β-nicotinamide adenine dinucleotide (β-NAD; 15 μg/ml) and hemin (15 μg/ml) in 5% CO_2 _for 18 h at 37°C. A portion of this culture was inoculated into fresh BHI broth supplemented with β-NAD and hemin at a concentration of 10% (vol/vol). The fresh culture was then incubated without shaking in 5% CO_2 _for 3 h at 37°C before infection.

### MIC

A routine agar dilution method was used to determine the MIC of the antibiotics [6]. The test strains were inoculated onto Muller-Hinton agar (Becton Dickinson and Company) supplemented with 0.5% yeast extract, 2% horse lysate instead of β-NAD and hemin in 5% CO_2 _for 18 h at 37°C.

### Cell culture model for *H. influenzae *infection

A549 cells (human lung carcinoma epithelial cell line, American Type Culture Collection CCL-185; Manassas) were cultured in Dulbecco's modified Eagle's medium (DMEM; Sigma-Aldrich, St. Louis, MO, USA) containing 10%(vol/vol) heat-inactivated fetal calf serum, penicillin (100 U/ml), and streptomycin (100 μg/ml). Cell cultures were seeded at a density of 1 × 10^5 ^cells per well in 24-well tissue culture plates with antibiotic-free culture medium 18 h prior to bacterial infection. The cell monolayers were rinsed three times with Hanks' balanced salt solution (HBSS; Sigma-Aldrich) to remove the antibiotics completely before bacterial infection.

For the invasion suppression assay of antibiotics, A549 cells were infected with NTHi at a multiplicity of infection (MOI) of 100 bacteria per host cell in the presence or absence (control) of 1 μg/ml of the antibiotic being tested (AMP, AZM, CLR, CFPN, and LVX). A mild centrifugal force (600 × g for 5 min) was applied to the 24-well tissue culture plates at the start of the infection period. After infection in 5% CO_2 _for 1 h at 37°C, the infected monolayers were gently washed three times with 1 ml HBSS to remove noninvasive bacteria, and incubated for 1 h in fresh medium containing gentamicin (GEN; 100 μg/ml) to kill the extracellular bacteria [2,7-9]. After incubation for 1 h, the monolayers were washed three times with HBSS and lysed by vigorous aspiration with 1 ml phosphate buffered saline (PBS; pH 7.4) containing 0.1% (wt/vol) sodium deoxycholate. The intracellular bacterial colony-forming units (CFU) were enumerated by diluting the lysed cells with PBS containing 0.01% (wt/vol) gelatin (BSG) [10] and plating them on the BHI agar (Becton Dickinson) supplemented with β-NAD and hemin using triplicate sampling of each infected well.

For the intracellular killing assay of antibiotics, after a 1-h treatment of GEN followed by a 1-h infection in the absence of antibiotics, the monolayers were washed three times with HBSS and were then further incubated with fresh culture medium in the presence or absence (control) of the antibiotic being tested in 5% CO_2 _for 2 h at 37°C. After an additional 2 h of incubation, the monolayers were washed three times with HBSS, and the intracellular bacteria were harvested to enable enumeration of the bacterial CFU, as described above.

### TEM

Infected cell monolayers on tissue culture inserts were fixed with a fixative composed of 1% glutaraldehyde and 4% formaldehyde in a 0.06 M phosphate buffer (pH 7.4). Samples were dehydrated in graded ethanol solutions, and embedded in Epon 812 mixture (Ladd Research Industries; Williston, VT, USA). Ultrathin sections were made with a LKB ultramicrotome (Pharmacia LKB, Uppsala, Sweden), stained with aqueous uranyl acetate and lead citrate, and analyzed with a JEOL 1200 EX-II transmission electron microscope (TEM; JEOL Ltd., Tokyo, Japan) at 80 kV as an accelerating voltage.

### Mouse model for *H. influenzae *infection

Four-week-old female C57BL/6J (*Slc11a*^s^, *H*-2^b^) mice (Charles River Japan, Yokohama, Japan) were injected intraperitoneally with cyclophosphamide at three days preinfection and on the day of infection at a dose of 200 mg/kg [11]. C57BL/6 mice were more susceptible than BALB/c (*Slc11a*^s^, *H*-2^d^) mice to *Psedomonas aeruginosa *infection in the agar-bead model [12]. The mice were intranasally inoculated with 20 μl of the bacterial suspension in BSG (1 × 10^9 ^CFU/mouse). Each mouse received intragastric administration of AMP, AZM, CLR, cefcapene pivoxil (CFPN-PI), or LVX (100 mg/kg/day) suspended in 0.5% (wt/vol) methylcellulose (400 cP). All antibiotic treatments except AZM were administered using a stomach sonde attached to a 1-ml syringe three times daily, while AZM was administered once daily for three days after infection. Control mice received intragastric administration of 0.5% methylcellulose three times daily for three days after infection. On day 4 after infection, the lung was removed, homogenized with BSG, and plated in order to determine the number of CFU [10,13-15].

### Statistics

Significant differences between the means plus or minus standard deviations (SDs) of different groups were examined using a two-tailed unpaired Student's *t *test. A P value of < 0.05 was regarded as statistically significant.

## Results

### *In vitro *susceptibility testing

The MICs of AMP, AZM, CLR, CFPN, LVX, and GEN against the six strains of NTHi are shown in Table 1. The MIC ranges of the antibiotics tested against the three BLNAR and the three BLNAS strains were as follows: BLNAR, AMP 1–4 mg/l; AZM 0.25–8 mg/l; CLR ≥ 8 mg/l; CFPN 1 mg/l; LVX ≤ 0.125 mg/l; and GEN 4 mg/l; BLNAS, AMP 0.25–0.5 mg/l; AZM 4–8 mg/l; CLR 8–16 mg/l; CFPN 0.063–0.125 mg/l; LVX ≤ 0.125 mg/l; and GEN 2–4 mg/l. It was clearly shown that LVX has high *in vitro *potency against both the BLNAR and BLNAS strains. Both AMP and CFPN also showed high potency against the BLNAS strains.

**Table 1:** *In vitro *potency of six antimicrobial agents against six clinical isolates of NTHi.

strain	MIC (μg/ml)						BLNAR or BLNAS
		
	AMP	AZM	CLR	CFPN	LVX	GEN	
YS001	4	8	>16	1	≤0.125	4	BLNAR
YS002	4	0.25	8	1	≤0.125	4	BLNAR
YS005	1	8	16	1	≤0.125	4	BLNAR
YS008	0.25	4	8	0.125	≤0.125	4	BLNAS
YS009	0.5	4	16	0.125	≤0.125	2	BLNAS
YS010	0.25	8	16	0.063	≤0.125	4	BLNAS

### Extracellular potency of antibiotics against *H. influenzae *entry into A549 cells

**Figure 1:**
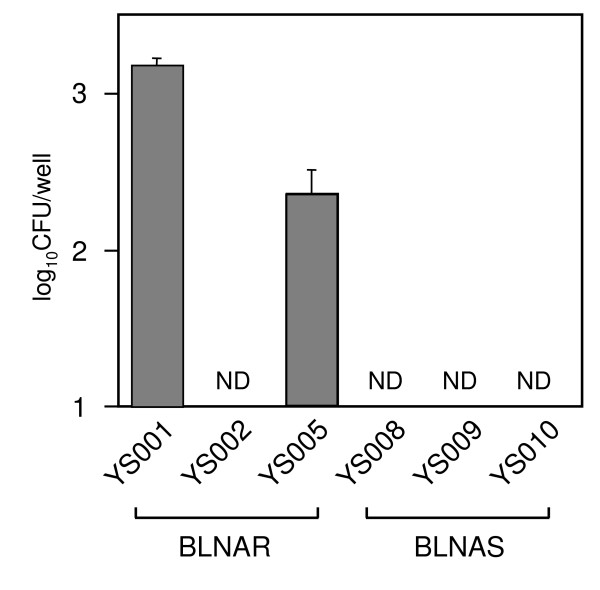
Entry of *H. influenzae *into A549 cells. Cell cultures (1 × 10^5 ^cells/well) were infected for 1 h at 37°C with 1 × 10^7 ^CFU of YS001, YS002, YS005, YS008, YS009, or YS010. At 1 h after infection, the cell culture medium was exchanged for fresh medium containing GEN (100 μg/ml). One hour later, the surviving intracellular bacteria were enumerated by plating. Data represent the means ± SD of three experiments with triplicate assays. ND, not detected.

**Figure 2:**
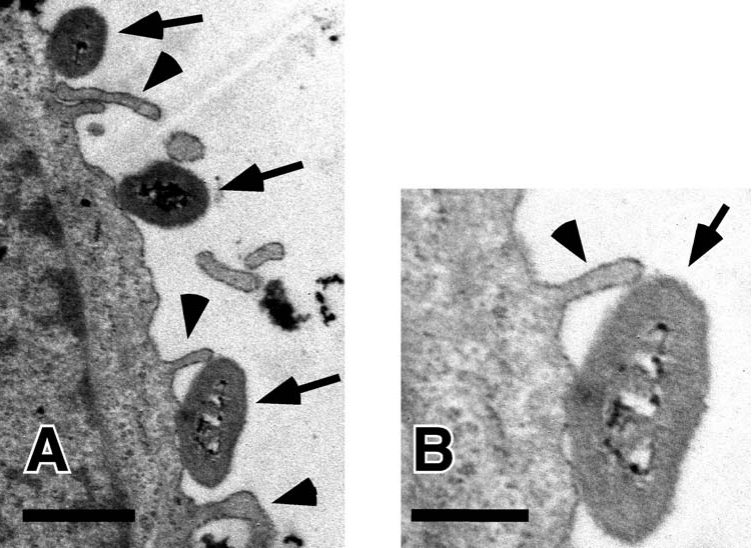
Electric microscopic observation of A549 cells infected with YS001. Bacteria attached (arrows) to the cell and the microvilli (arrow heads) of the cell are recognized. Bars, 1 μm (A) and 0.5 μm (B).

**Figure 3:**
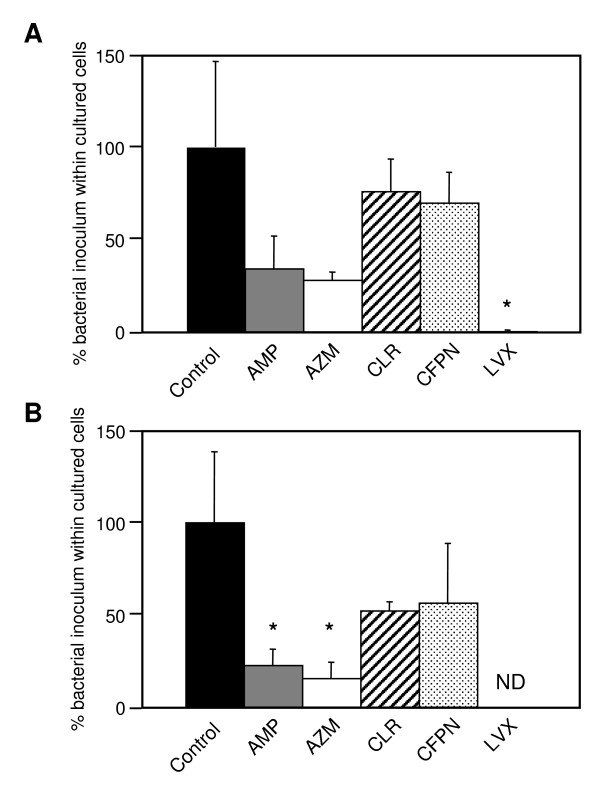
The invasion suppression assay of antibiotics. A549 cells (1 × 10^5 ^cells/well) were infected for 1 h at 37°C with 1 × 10^7 ^CFU of YS001 (A) or YS005 (B) in the presence or absence (control) of AMP, AZM, CLR, CFPN, or LVX (1 μg/ml). At 1 h after infection, the cell culture medium was exchanged for fresh medium containing GEN (100 μg/ml). One hour later, surviving intracellular bacteria were enumerated by plating. Data represent the means ± SD of three experiments with triplicate assays. *, *P *< 0.05 (compared with control). ND, not detected.

We first examined the entry level of NTHi strains into A549 cells using a GEN protection assay. Although there was somewhat less variability in invasiveness among strains, two (YS001 and YS005) of the six tested strains were considered to be invasive strains (Fig. 1). It was reported previously in TEM analyses and confocal macroscopic studies that NTHi can initiate cytoskeletal rearrangement within the human airway epithelium, resulting in the internalization of the bacteria within nonciliated human airway epithelial cells by the process of macropinocytosis [16]. Using TEM analysis, we detected the microvilli of A549 cells surrounding organisms, as shown in Fig. 2. Therefore, we assume that some but not all NTHi strains are able to enter epithelial cells by the process of macropinocytosis. We next examined the inhibitory effects of each antibiotic on the entry and replication of YS001 and YS005 in A549 cells. The cells were infected with either YS001 or YS005 (MOI = 100) for 1 h at 37°C in the cell culture medium supplemented with each antibiotic (AMP, AZM, CLR, CFPN, or LVX; 1 μg/ml) or in cell culture medium that was not supplemented. Figure 3 shows that the LVX treatment was the most effective of the antibiotics tested in the present study in suppressing the entry of every strain into this line of cells. AMP or AZM treatment decreased the number of intracellular bacteria of each strain by 70–85%, while CLR or CFPN treatment decreased the number of bacteria by 30–50%. These results indicate that the ranking of the treatments in terms of their efficacy for suppressing the entry of invasive BLNAR strains was LVX>AMP = AZM>CLR = CFPN.

### Intracellular potency of antibiotics against *H. influenzae *within A549 cells

**Figure 4:**
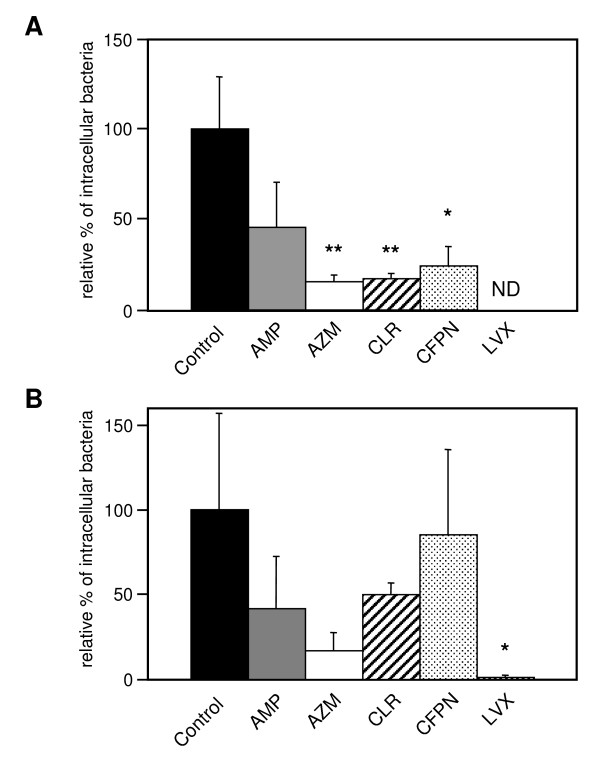
Intracellular killing assay of antibiotics. A549 cells (1 × 10^5 ^cells/well) were infected for 1 h at 37°C with 1 × 10^7 ^CFU of YS001 (A) or YS005 (B) in the absence of antibiotics. After a 1-h infection followed by a 1-h incubation period with GEN (100 μg/ml), the cell culture was incubated in the presence or absence (control) of AMP, AZM, CLR, CFPN, or LVX (1 μg/ml). Two hours later, the surviving intracellular bacteria were enumerated by plating. Data represent the means ± SD of three experiments with triplicate assays. *, *P *< 0.05 (compared with control). **, *P *< 0.01 (compared with control). ND, not detected.

After infection with YS001 or YS005 for 1 h at 37°C in the cell culture medium without supplementation of any antibiotic followed by subsequent incubation for 1 h in fresh medium supplemented with GEN to kill extracellular bacteria, the infected monolayers were further incubated with fresh culture medium supplemented with each antibiotic (AMP, AZM, CLR, CFPN, or LVX; 1 μg/ml) or not supplemented for 2 h at 37°C. Figure 4 shows that the LVX treatment was the most effective in reducing the number of intracellular bacteria of every strain. In contrast, intracellular bactericidal activities brought about by treatment with the other four antibiotics were detected in each strain, but to different degrees. The intracellular bactericidal activity against invasive BLNAR strains of the tested antibiotics can be ranked as follows: LVX>AZM>AMP = CLR = CFPN.

### Mouse model of intranasal infection with *H. influenzae*

To date, mouse models of *H. influenzae *pneumonia have been rarely reported since it is difficult for this bacterium to infect mice [17,18]. Therefore, we pursued a new mouse model of NTHi lung infection in order to evaluate the efficacy of the antibiotics tested. In the present study, we used 4-week-old C57BL/6 mice. Generally, young immature mice are more susceptible to bacterial infection than fully grown mice [19]. In addition, the mice were pretreated intraperitoneally with cyclophosphamide to induce granulocytopenia [20]. We previously observed a decrease of at least 90% in peripheral blood granulocytes after cyclophosphamide treatment [11]. When cyclophosphamide-pretreated mice were intranasally inoculated with 1 × 10^9 ^CFU of NTHi, the bacteria continuously resided in the lungs of the mice at more than 10^5 ^CFU when monitored for up to four days after inoculation (data not shown).

### Efficacies of antibiotics against *H. influenzae *colonization in mouse lung

**Figure 5:**
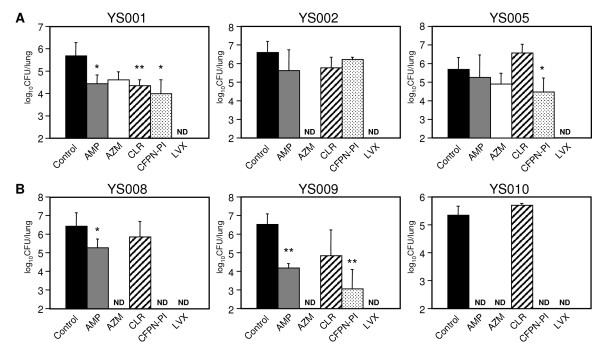
Bacterial clearance by antibiotic treatment in the lung. Mice infected intranasally with YS001, YS002, YS005, YS008, YS009, or YS010 were intragastically administered AMP, AZM, CLR, CFPN-PI, LVX (100 mg/kg/day), or methylcellulose (control) once (AZM) or three times (AMP, CLR, CFPN-PI, LVX, and methylcellulose) daily for three days after infection. On day 4 after infection, the mice were harvested, and bacterial loads were determined in lung homogenates. Data represent the means ± SD of five mice per group. *, *P *< 0.05 (compared with control). **, *P *< 0.01 (compared with control). ND, not detected.

We evaluated the efficacy of the antibiotics for reducing the colonization of NTHi in the mouse lung. Based on the data from previous reports [21-24] and the suppliers' instructions, we estimated peak antibiotic concentrations of 1 to 2 μg/ml (AZM and CFPN-PI), 2 to 3 μg/ml (AMP and LVX), or 5 to 6 μg/ml (CLR) in the blood of mice from 30 to 120 min after intragastric administration. Figure 5 shows that the intragastric administration of LVX was extremely effective against both the BLNAR and BLNAS strains of NTHi. The AZM treatment was also extremely effective for the complete eradication of BLNAS strains from the lung, while the CLR treatment showed no efficacy. Treatment with AMP or CFPN-PI was also highly effective against BLNAS infection. However, except for LVX, none of the tested antibiotics was found to be effective against invasive BLNAR strains.

## Discussion

NTHi strains are not traditionally considered to be invasive [1,25]; however, recent works have shown that NTHi can penetrate human respiratory epithelial cell lines [4,7] and primary human airway epithelial cells [16]. In addition, some reports have shown that NTHi can reside intracellularly in human tissue samples [3,9,26]. In the present study, two of the three BLNAR strains examined certainly entered the A549 human lung epithelial cell line (Fig. 1). Nevertheless, in the mouse infection model, there were no differences in the infection doses of bacteria in the lung homogenates between BLNAR and BLNAS or between invasive and noninvasive strains (Fig. 5). To date, we have been unable to detect intracellular bacteria in lung sections by TEM analysis after infection with NTHi.

LVX showed potent *in vitro *antibacterial activity against both the BLNAR and BLNAS strains (Table 1), and was found to be overwhelmingly effective against invasive BLNAR strains in the cell culture infection model (Figs. 3 and 4). After 1-h incubation in 5% CO_2 _at 37°C, LVX used at a concentration of 1 μg/ml (≥ 8 times the MIC) killed about 99% of bacteria (both YS001 and YS005) in the cell culture medium; still, as many as 1 × 10^5 ^CFU per well of extracellular bacteria were present after the LVX treatment (data not shown). The 1 × 10^5 ^CFU of live bacteria was surplus to the amount required for entering the cells, since YS001 and YS005 were able to enter the cells with 1.3 × 10^3 ^CFU and 2.4 × 10^2 ^CFU, respectively (Fig. 2). Consequently, LVX appears to have an extracellular potency to suppress the invasion of bacteria, since almost no bacterium was detected within the cells after the LVX treatment (Fig. 3). By comparison, it seems that AMP, AZM, or CLR used at lower concentrations of 1/4, 1/8, or 1/16 of MIC, respectively, all have extracellular potency to suppress the invasion of BLNAR strains into A549 cells, as shown in Fig. 3. We have previously reported that a sub-MIC of AZM can reduce the invasive activity of *Salmonella enterica *serovar Typhimurium into the Henle-407 human intestinal epithelial cell line [10]. Presumably, then, a sub-MIC of each antibiotic might affect the expression of the bacterial invasion apparatus directly and/or indirectly. Fluoroquinolones have long been known to accumulate in eukaryotic cells. The cellular concentrations of fluoroquinolones are generally 4- to 10-fold higher than the extracellular concentrations [27]. In contrast, up to now, all studies have reported a lack of accumulation (i.e., an apparent intracellular concentration lower than the extracellular concentration at equilibrium) for all β-lactams [27].

In sharp contrast with β-lactams, macrolides show a marked intracellular accumulation in almost all cells. The cellular concentrations of CLR and AZM are 10- to 20-fold and 40- to 300-fold larger, respectively, than the extracellular concentrations [27]. Still, it seems that every antibiotic can reduce the intracellular CFU of invasive BLNAR strains to some extent, as shown in Fig. 4. Accordingly, intracellular bacteria are exposed to various killing actions of the cells. Most obligate and facultative intracellular bacteria have mechanisms for escaping from the killing systems inside the cells. In contrast to such bacteria, the response of NTHi to the defense system inside the cells remains unclear; however, intracellular NTHi must suffer a synergy effect between the activity of the antibiotic and the killing actions of the cells. Moreover, we must also consider the fact that antibiotics may exert either favorable or unfavorable action on the host cells, which in turn modulate the bactericidal activity of the antibiotics [27].

*H. influenzae *is a strict parasite of humans which efficiently colonizes the upper respiratory tract [1]. When constructing the mouse model of intranasal infection with NTHi, we considered using the ICR, ddY, and C57BL/6 mouse strains. As a result of the preliminary experiments, intranasally inoculated bacteria were detected in the lungs of cyclophosphamide-pretreated C57BL/6 mice, while the inoculated bacteria were not detected in the lungs of cyclophosphamide-pretreated ICR or ddY mice. On the one hand, C57BL/6 mice carry the *Nramp1*^*D169 *^(natural resistance-associated macrophage protein 1 gene; new nomenclature, *Slc11a1*) allele (a point mutation at nucleotide position 596, resulting in a glycine-to-aspartate substitution) in the genetic background. On the other hand, ICR and ddY mice carry the *Nramp1*^*G169 *^gene. A single locus (*Nramp1*) that is mapped to the proximal region of mouse chromosome 1 has been shown to influence the early phase of bacterial replication in reticuloendothelial cells [28]. The expression of *Nramp1 *in mice confers innate resistance to certain bacterial infections such as *Mycobacterium bovis *(BCG), *Leishmania donovani*, and *S. enterica *serovar Typhimurium [29,30]. Therefore, C57BL/6 mice are susceptible to infection with these bacteria. It is possible that the expression of *Nramp1 *in mice would also confer innate immunity to infection with *H. influenzae*, so NTHi could colonize in the lungs of C57BL/6 mice after intranasal infection.

It has been reported that in Japanese and U.S. clinical isolates, the prevalence rates of each resistance class of *H. influenzae *in Japan (n = 296) and the United States (n = 100) are, respectively, 55.1% and 46% for BLNAS; 26.4% and 13% for low-BLNAR with a low degree of AMP resistance; 13.2% and 0% for BLNAR; 3% and 36% for TEM-1 type and ROB-1 type β-lactamase-producing AMP-resistant *H. influenzae *(BLPAR); and 2.4% and 3% for Hib strains [31]. These data indicate a worldwide increase of AMP-resistant *H. influenzae *isolates. Therefore, it seems likely that the use of fluoroquinolones or macrolides for the oral treatment of respiratory tract infections associated with *H. influenzae *should be useful. In fact, fluoroquinolones have been widely used in the treatment of respiratory tract infections, and their use has increased extensively in hospitals and long-term care facilities [32]. However, it has also been reported that the emergence of *H. influenzae *isolates with reduced susceptibility to fluoroquinolones is becoming a clinical and public health concern [33-35]. Under these circumstances, the new guidelines in the context of the risk management of community-acquired pneumonia in adults are the following: Young and otherwise healthy patients who can be safely treated as outpatients will usually respond to AZM. Doxycycline should be used with caution because of increasing resistance to this agent, and reserved for use when other options are not available. Older patients, or those with substantial comorbidities, will usually respond well to combinations of a β-lactam agent (such as high-dose amoxicillin/clavulanate) plus a macrolide. Newer generation recommended (so-called "respiratory") fluoroquinolones can be used as first-line agents but should be reserved for use in higher risk or drug-intolerant patients in order to slow the emergence of resistance to this class of drugs [36]. Therefore, in spite of the fact that the oral LVX treatment showed excellent therapeutic efficacy in the mouse model of intranasal infection with NTHi compared with other antibiotic treatments (Fig. 5), fluoroquinolones should be avoided or used with great caution. As another option, combinations of a β-lactam agent plus a macrolide could be expected to have a modest therapeutic benefit in the oral treatment of respiratory tract infections associated with *H. influenzae*, which we will examine in future studies.

Miyazaki *et al*. have demonstrated that although the MICs of AZM and CLR are similar for three strains of *H. influenzae *(BLNAR, BLNAS, and BLPAR) at a dose of 50 mg/kg, the area under the concentration curve and the half-life of AZM in the lungs are 3-fold higher and 6-fold longer, respectively, than those of CLR in mice. Thus, the authors concluded that AZM may be useful for both AMP-susceptible and AMP-resistant bronchopneumonia caused by *H. influenzae *infections [5]. The present results support their conclusion. AZM has potent antibacterial activity against BLNAS strains in mice compared with CLR (Fig. 5). It is well known that since AZM, the only azalide macrolide antibiotic, has the advantage of an extensive half-life of approximately 68 h, it is possible to administer it once a day for a short duration while sustaining effective serum and tissue concentrations for at least 10 days [37]. The intracellular (i.e., within leukocytes and fibroblasts) and tissue concentrations of AZM make it an excellent option for intracellular pathogens [37]. The dose and frequency of the oral administration of each antibiotic used in the present study in the treatment of a 70 kg adult human as recommended by the suppliers are as follows: AMP, 250–500 mg, four to six times daily; AZM, 500 mg once daily; CLR, 200 mg, twice daily; CFPN-PI, 100 mg, three times daily; and LVX, 100 mg, three times daily. Therefore, the dose for the oral administration of each antibiotic in mice in the present study was set at 100 mg/kg daily, which is thought to be two to 23 times more than sufficient to produce effective antibacterial activity. Nevertheless, with the exception of LVX, we found no antibiotics that were effective against invasive BLNAR strains (YS001 and YS005) in the mouse infection model (Fig. 5). We hope that our future studies on invasive BLNAR strains using the mouse infection model will identify effective antibiotics for human medical use.

## Conclusion

In summary, fluoroquinolone LVX had potent activities against both the process of invasion into the A549 human lung epithelial cell line and the residence in the cell line of invasive BLNAR strains of NTHi, as compared with AMP, AZM, CLR, and CFPN. For the mouse model of infection with NTHi, four-week-old female C57BL/6 mice were pretreated intraperitoneally with cyclophosphamide to induce granulocytopenia before intranasal infection with NTHi. We evaluated the efficacy of the antibiotics at reducing the colonization of NTHi in the mouse lung. The results showed that LVX was extremely effective in treating both the BLNAR and BLNAS strains of NTHi as compared with AMP, AZM, CLR, and CFPN-PI. In particular, with the exception of LVX, there were no antibiotics that were effective against invasive BLNAR strains in the mouse infection model. These observations provide evidence that fluoroquinolones have great bactericidal activities against quinolone-susceptible NTHi strains. Further studies are needed to identify effective antibiotics for human use other than fluoroquinolones as an initial treatment that provides coverage of both BLNAR and BLNAS or invasive and noninvasive strains of NTHi.

## Abbreviations

AMP: Ampicillin; AZM: Azithromycin; BLNAR: β-lactamase-negative AMP-resistant *Haemophilus influenzae*; BLNAS: β-lactamase-negative AMP-susceptible *Haemophilus influenzae*; BLPAR: β-lactamase-producing AMP-resistant *Haemophilus influenzae*; BSG: Phosphate-buffered saline containing 0.01% (wt/vol) gelatin, pH7.4; NTHi: Nonencapsulated and nontypeable *Haemophilus influenzae*; CFPN: Cefcapene; CFPN-PI: CFPN pivoxil; CFU: Colony-forming units; CLR: Clarithromycin; DMEM: Dulbecco's modified Eagle's medium; GEN: Gentamicin; HBSS: Hanks' balanced salt solution; LVX: Levofloxacin; MIC: Minimum inhibitory concentration; MOI: Multiplicity of infection; NAD: Nicotinamide adenine dinucleotide; PBS: Phosphate-buffered saline, pH7.4; SD: Standard deviation; TEM: Transmission electron microscope.

## Competing interests

The author(s) declare that they have no competing interests.

## Authors' contributions

YS and HM carried out most of the experiments; ME helped with the cell culture model of the infection experiments; MN helped with the TEM analysis; KU supplied the NTHi strains and provided advice on the experiments and manuscript; SO provided advice on the experiments and manuscript; HM designed all the experiments and prepared the manuscript.

## Pre-publication history

The pre-publication history for this paper can be accessed here:


